# Optocollic responses in adult barn owls (*Tyto furcata*)

**DOI:** 10.1007/s00359-021-01524-z

**Published:** 2021-11-23

**Authors:** Hermann Wagner, Ina Pappe, Hans-Ortwin Nalbach

**Affiliations:** 1 Max-Planck-Institut für Biologische Kybernetik, Max-Planck-Ring 11, 72076 Tübingen, Germany; 2 Present Address: Institut für Biologie II, RWTH Aachen, Worringerweg 3, 52074 Aachen, Germany; 3 Universitätsklinik für Anaesthesiologie, Waldhörnlestrasse 22, 72072 Tübingen, Germany

**Keywords:** Nystagmus, Optokinetic, Optomotor, Saccade, Binocular

## Abstract

Barn owls, like primates, have frontally oriented eyes, which allow for a large binocular overlap. While owls have similar binocular vision and visual-search strategies as primates, it is less clear whether reflexive visual behavior also resembles that of primates or is more similar to that of closer related, but lateral-eyed bird species. Test cases are visual responses driven by wide-field movement: the optokinetic, optocollic, and optomotor responses, mediated by eye, head and body movements, respectively. Adult primates have a so-called symmetric horizontal response: they show the same following behavior, if the stimulus, presented to one eye only, moves in the nasal-to-temporal direction or in the temporal-to-nasal direction. By contrast, lateral-eyed birds have an asymmetric response, responding better to temporal-to-nasal movement than to nasal-to-temporal movement. We show here that the horizontal optocollic response of adult barn owls is less asymmetric than that in the chicken for all velocities tested. Moreover, the response is symmetric for low velocities (< 20 deg/s), and similar to that of primates. The response becomes moderately asymmetric for middle-range velocities (20–40 deg/s). A definitive statement for the complex situation for higher velocities (> 40 deg/s) is not possible.

**Supplementary Information:**

The online version contains supplementary material available at 10.1007/s00359-021-01524-z.

## Introduction

Birds and mammals share a similar anatomical forebrain organization (Stacho et al. 2020). This is reflected in cognitive behavior of, for example, owls and crows rivalling those of primates (Orlowski et al. 2018; Zahar et al. 2018; Nieder et al. 2020). If anatomy and cognition of birds are similar to that of mammals, one may speculate that “simpler”, reflex-like behavior might even more resemble mammalian, including human, behavior. Test cases for this claim are the optokinetic (OKR), the optocollic (OCR), and the optomotor (OMR) responses. These reflexes help to stabilize the visual world via movement of eyes (OKR) or head (OCR), or serve course-control (OMR) (Carpenter 1988; Huang and Neuhaus 2008; Masseck and Hoffmann 2009). Almost all animals show one or several of these reflexes—depending on eye-movement capability and state of activity (Gioanni 1988). These reflexes are typically elicited by moving a highly structured visual surround across the visual field of the observer mimicking his self-movement in a stationary world. A classic example occurs when one sits in a train and the train on the next platform starts to move. A slow-phase segment, during which the subject follows the movement of the wide-field stimulus, and fast return saccades characterize the reflexes. This leads to a sawtooth-like pattern of gaze called nystagmus. For a long time, OCRs, OKRs and OMRs were studied in a broad variety of animals [e.g. flies (Borst et al. 2010), crabs (Sandeman et al. 1975; Nalbach 1989; Barnatan et al. 2019), goldfish (Easter 1972; Masseck et al. 2010), frogs (Dieringer and Precht 1982), geckos (Masseck et al. 2008), turtles (Ariel 1997), pigeon (Gioanni et al. 1981; Gioanni 1988; Nalbach 1992; Türke et al. 1996; Maurice et al. 2006), chicken (Wallman and Velez 1985), hummingbirds (Goller and Altshuler 2014; Gaede et al. 2016), cat (Schweigart and Hoffmann 1988), ferret (Hupfeld et al. 2007), monkeys (Cohen et al. 1977; Lappe et al. 1998; Distler et al. 1999), and humans (van den Berg and Collewijn 1988)]. Recent work has focused on model systems like zebrafish, mouse, and healthy as well as impaired human subjects (e.g. Dieterich et al. 2009; Huang and Neuhauss 2008; Naumann et al. 2016; Agarwal et al. 2016; Kretschmer et al. 2017; Lappi et al. 2020). A quantitative behavioral study on owls is missing. We only found a brief qualitative mentioning of OMRs in three owl species, not including barn owls, in Tauber and Atkin (1968).

We worked with barn owls (*Tyto furcata*). When we speak of “owls” in the following, we refer to barn owls, if not stated otherwise. Owls represent an interesting case as their frontally oriented eyes create a large binocular overlap that allows the owls to extract depth by stereo vision (Willigen et al. 1998, 2002, 2003). These birds have a well-developed scleral ring that stabilizes the eyes in the skull (Franz-Odendaal and Krings 2019). Moreover, owls have very large, elongated eyes. The eyes are rather fixed in the skull, and these birds cannot move their eyes more than one to four degrees (Steinbach and Money 1973; Du Lac and Knudsen 1990; Nieder and Wagner 2000; Iwaniuk et al. 2008; Netser et al. 2010). Owls exhibit OCRs to stimulation with visual wide-field patterns. This is similar to the other birds mentioned before. However, most other bird species as well as e.g. frogs, turtles and many mammals have laterally-positioned eyes, and exhibit so-called asymmetric OKRs or OCRs, while primates have frontally-positioned eyes and have a symmetric horizontal OKR. Symmetry or asymmetry of the reflexes occurs under monocular stimulation, when nasal to temporal (N–T) and temporal to nasal (T–N) directions of movement may be discriminated. Lateral-eyed vertebrates typically exhibit a higher gain (for a definition, see Eq. 1 below) when stimulated in the T–N than in the N–T direction (e.g. Gioanni et al. 1981; Dieringer and Precht 1982; Wallman and Velez 1985). By contrast, the frontal-eyed primates show similar gains in both stimulus directions (e.g. van den Berg and Collewijn 1988; Distler et al. 1999). Thus, the question arises whether the OCRs of owls more closely resemble those of their avian relatives or of primates with their similar visual world.

To study this issue, we tested barn owls in binocular and monocular settings. We show here that adult barn owls exhibit an OCR not quite as symmetric as the OKR in primates, but far less asymmetric than in the chicken.

## Materials and methods

Six tame, hand-raised owls (codes: G, H, I, J, K, L) participated in the experiments. Owls start to fly between 50 and 60 days of age, and soon after, they have to catch prey by themselves. Shawyer (1998) reports that adult feather length is, on average, achieved at postnatal day 67. Thus, we use the term 'adult' for fledged birds being older than 67 days.

### Set-up and stimuli

**Fig. 1:**
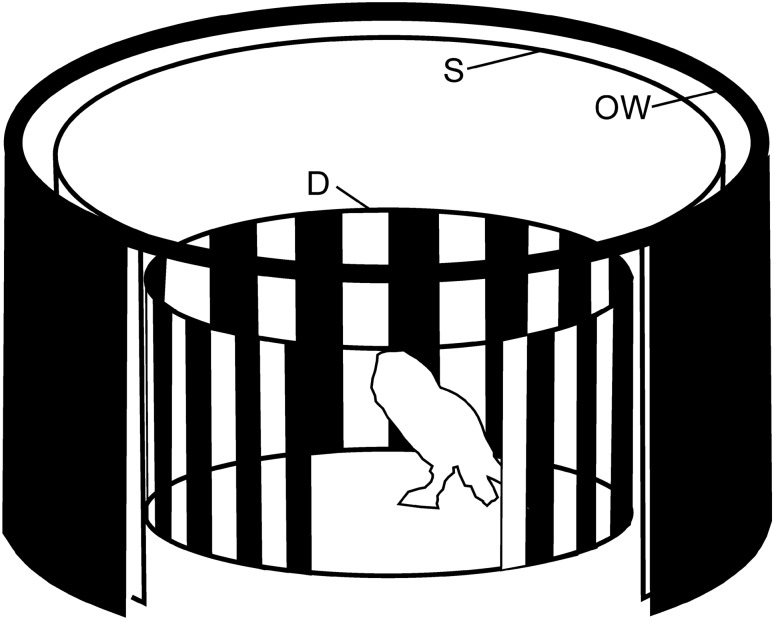
The stimulus drum. We used the same set-up as Türke et al. (1996). The sketch shows the animal in the center of the rotating drum with a vertically striped pattern (*D*). For homogeneous illumination, the outer stationary cylinder (OW) carried 72 equally spaced light bulbs (not shown) whose light was diffused by an opaque screen (*S*). For further details, see text

Visually induced optocollic reactions were measured with a rotating drum (Fig. 1; for details see also Türke et al. 1996). The drum (diameter 64 cm, height 46 cm, angle subtended in elevation 70°) carried the stimulus pattern. We used two high-contrast wide-field stimuli: (1) evenly horizontally and vertically spaced squares (2.7° × 2.7° as seen from the center of the drum) (Nalbach 1992), and (2) a white-and-black striped pattern (horizontal wavelength 10° as seen from the center of the drum) (Fig. 1). A DC-driven motor rotated the drum, and thus the pattern, at constant velocities (see below). A potentiometer attached to its shaft monitored the rotation. The pattern was diffusely illuminated from outside. The average light intensity was 27.3 cd/m^2^.

**Fig. 2:**
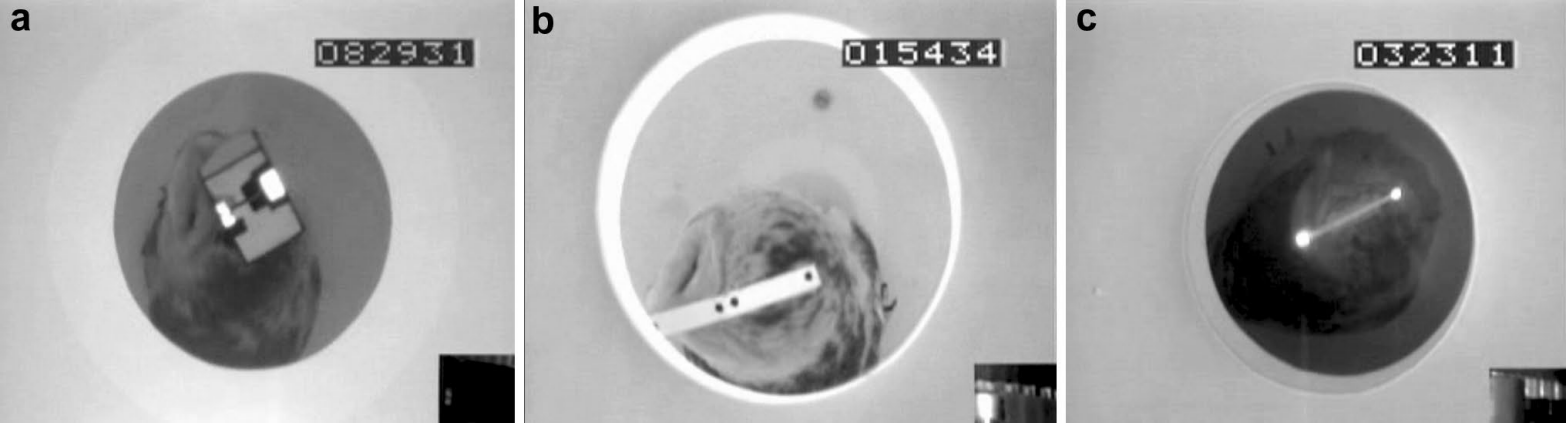
**a**, **b** The animal, sitting in the drum as seen from the position of the camera. Position markers appear as white (**a**) or black dots (**b**). Two different types of eye occluders are shown. **c** A faintly visible animal with the high contrast reflecting spots that facilitated reconstruction of head movement. Note also the gearwheel in the lower right corner that allowed visual control of drum rotation

During an experiment, the animal was sitting on a perch, positioned in the middle of the drum, with its legs loosely fastened to the perch by a ribbon made of leather. The long axis of the perch was defined as perpendicular to zero azimuth in an external coordinate system. Thus, if the owl was sitting in normal posture, its view centered at zero azimuth. Sheets of paper screened the bottom and top of the drum. The sheets masked stationary contours so that the reaction of the animals corresponded to a “stare” or “delayed” OCR (for details see Türke et al. 1996). Videotaping of the owl’s head from above was possible through a 12 cm-wide circular hole in the center of the top of the inner drum (Fig. 2).

### Data recording

Recording of monocular and binocular OCRs took place between February 1992 and May 1993. A recording session never lasted longer than one hour. For recording monocular OCRs, either the right or the left eye of a bird was occluded (Fig. 2a). Different eye covers were tested. All worked similarly well. The eye cover was fixed to a holder that had been cemented to the animal's skull under anesthesia [for further details on surgery and anesthesia see Wagner (1993)]. The surgery and the experiments were carried out under a permit issued by the Regierungspräsidium Tübingen, Germany. Recording gear was mounted shortly before an experiment and removed immediately afterwards.

Optocollic reactions were recorded without earlier training. Our goal was to record data at different drum velocities (5, 8, 10, 15, 20, 30, 40, 60, 80, 93 deg/s) that were presented in a pseudo-random order, clockwise (cw) and counter-clockwise (ccw) rotation alternating. Drum velocities of 8, 80, and 93 deg/s were only used for monocular stimulation, while data for the other seven stimulus velocities were recorded for both monocular and binocular stimulation. Because of the owl's restricted eye-movement capability mentioned above, we recorded only head rotations. Position markers were drawn on the eye cover (Fig. 2a; video in supplements) or a stripe of paper that was fixed to the holder and/or to the feathers on top of the owl’s head (Fig. 2b). Alternatively, a stripe of cardboard with two reflection spots was fixed to the feathers on top of the head of the owl (Fig. 2c). The stripe was not moving relative to the head as assured by visual inspection. The reflection spots were illuminated via an infrared light source and videotaped from above (Fig. 2c).

### Data analysis

Automatic analysis of the video image took place off-line by stepping the video recorder forward by a preset number of frames. The typical temporal resolution was 80 ms, but could be higher for high velocities and lower for low velocities. The frame was grabbed with a videoboard (FG 100, Imaging Technology, Inc.), and transferred into the main memory of a PC. In this way, the projection of the position markers onto the horizontal plane was imaged. After contrast-enhancement and contrast clipping, the position of the position markers was automatically digitized and written into computer memory. Likewise, the voltage of the potentiometer was stored in synchrony. From these readings, the azimuthal orientation of the owl’s head and the azimuthal position of the pattern were derived and stored for further processing. The horizontal angular velocity of the head was calculated from head orientation. The beginning and the end of slow-phase segments were determined by a thresholding mechanism (for details see Türke et al. 1996). The results were controlled later by visual inspection and corrected, if necessary.

During a slow-phase segment, the owl followed the moving pattern by head rotation. In such a closed-loop situation, the stimulus that elicits the slow phase of the OCR is the retinal-slip speed in the animal’s perception (Türke et al. 1996). Note that we could not measure this directly. We could only determine the difference between the angular velocity of the external stimulus as derived from the potentiometer data and the angular velocity of the head. It needs to be kept in mind that the potentiometer data need not contain all information that the animal uses for its perception (see also Discussion). Similarly, we calculated the gain that characterizes the effectiveness of the OCR from the angular velocity of the stimulus as derived from the potentiometer data. We define the “closed-loop gain” as

1 $${\text{gain}}\left( \% \right) = \frac{{{\text{angular velocity of animal's head}}}}{{{\text{angular velocity of stimulus}}}} \times 100$$

The gain was determined from the mean angular velocity of both the animal’s head and the stimulus during each single slow-phase segment. In other words, one slow-phase segment provided one data point for the analysis. We analyzed only slow-phase segments having a duration of a least five data points.

We also determined the durations and the amplitudes of the slow-phase segments. The duration of a slow-phase segment is the time from the beginning (after the return saccade) to the end (before the return saccade starts) of the following response in seconds. The amplitude (in degrees) of a given slow-phase segment is the product of duration and the mean angular velocity of the owl’s head during the respective slow-phase segment.

### Statistics

Most of our data did not show normal distributions (see below). Parametric analyses were not adequate in these cases. Therefore, we used nonparametric statistics, specifically the Mann–Whitney *U* test to analyze the difference of two not paired samples. Some data sets were also subjected to a correlation analysis.

## Results

**Fig. 3:**
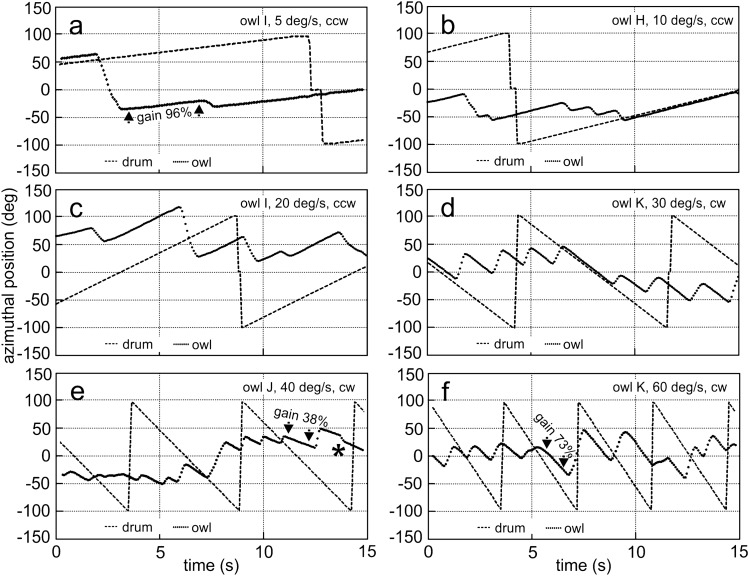
Examples of binocular OCRs of adults. Different velocities: **a** 5 deg/s, **b** 10 deg/s, **c** 20 deg/s, **d** 30 deg/s, **e** 40 deg/s, **f** 60 deg/s, different directions (ccw: **a**–**c**; cw: **d**–**f**), and different owls (I: **a**, **c**; H: **b**; K: **d**, **f**; J: **e**). Gain values, indicated for selected slow-phase segments in **a**, **e**, and **f**, provide quantitative information. The asterisk in **e** points a fast turn in the direction of stimulus movement. Dashed lines represent a reference position on the wide-field pattern, plotted in the range between ± 100°. Note that the dashed lines between + 100° and − 100° and the sawtooth-like appearance of stimulus position are due to wrapping. Filled circles signify the position of the owl’s head in azimuth

Although barn owls are able to actively rotate their head by more than 270° (Krings et al. 2017), we typically observed a range of ± 50° during the slow-phase movements, with some extreme head rotations beyond 100° (Fig. 3). The slow-phase movements were interrupted by reset phases (return saccades) in the opposite direction. Typically, the return saccades had a higher head-turning velocity than the slow-phase movements (Fig. 3).

In total, we analyzed 118 sequences, containing 1234 slow-phase segments. Binocular responses were obtained from five birds (owls G, H, I, J, K), providing 387 slow-phase segments for analysis. Monocular data consisted of 847 slow-phase movements that were collected from the same five birds from which we obtained binocular data and owl L for which no binocular data were recorded.

**Fig. 4:**
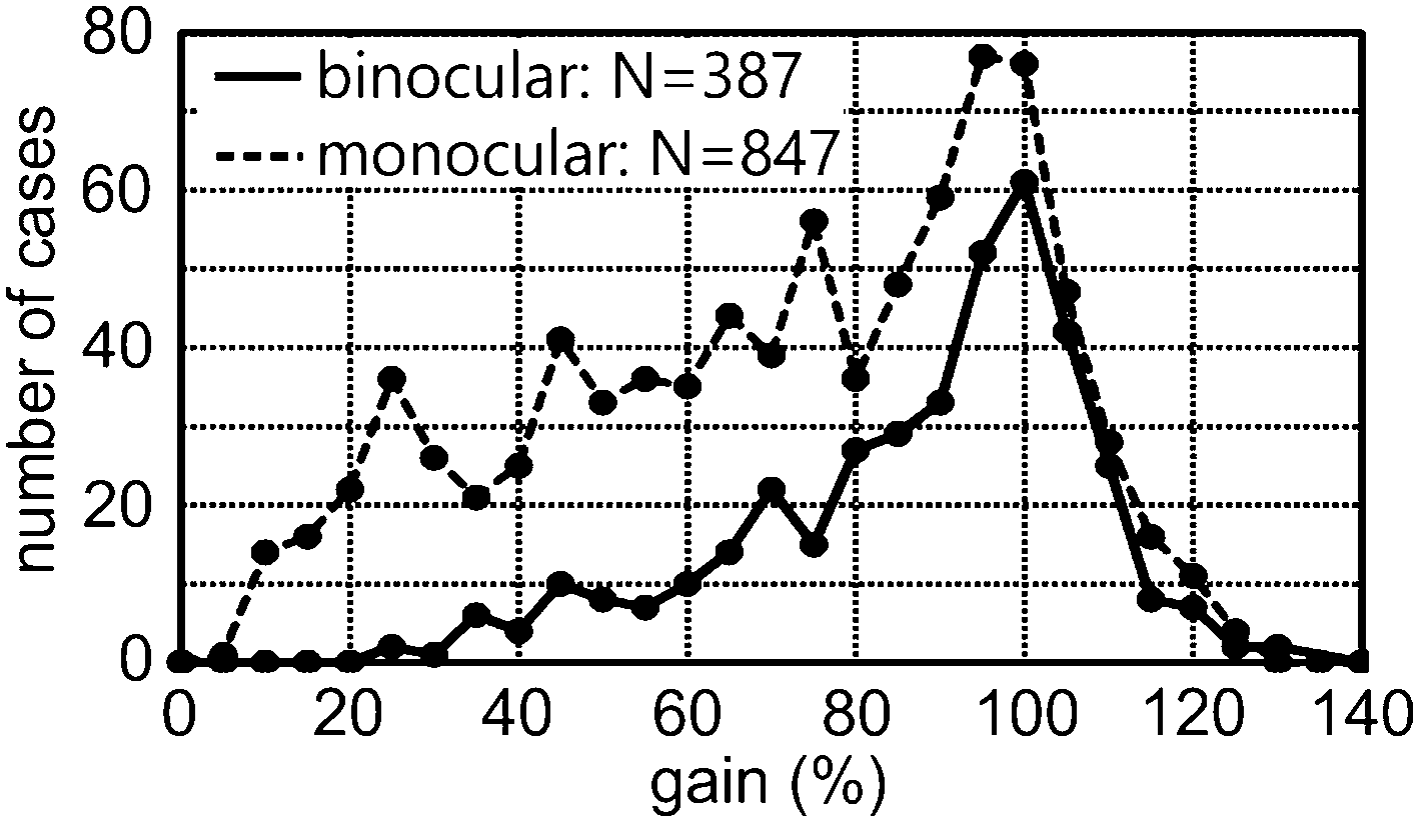
Distributions of gains. The distributions of binocular and monocular gains across all velocities are shown. N specifies the number of slow-phase segments in each condition. Note the maximum value close to 100%, and the skewed distribution with a long tail towards 0% gain, and a short tail with gains > 100%

**Fig. 5:**
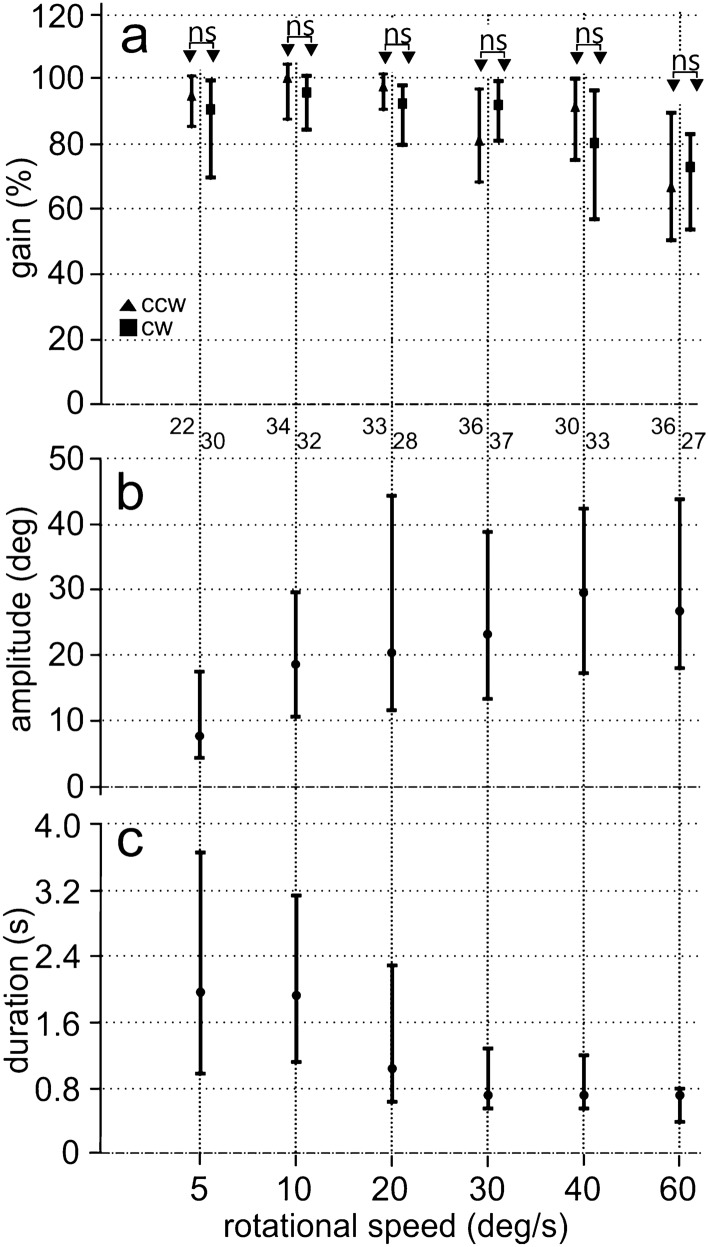
Specific characteristics of binocular OCRs. **a** Gains for clockwise (cw) and counter-clockwise (ccw) stimulation. The gains are not statistically significantly different (“ns”). **b** The dependence of the turning amplitude on the stimulus velocity. **c** The dependence of the duration of the slow-phase segments on the stimulus velocity. The turning amplitude increases, while the slow-phase duration decreases with stimulus velocity (for a quantitative analysis see text). Shown are the median values and the first and third quartiles. The numbers below **a** specify the number of cases for each condition

**Fig. 6:**
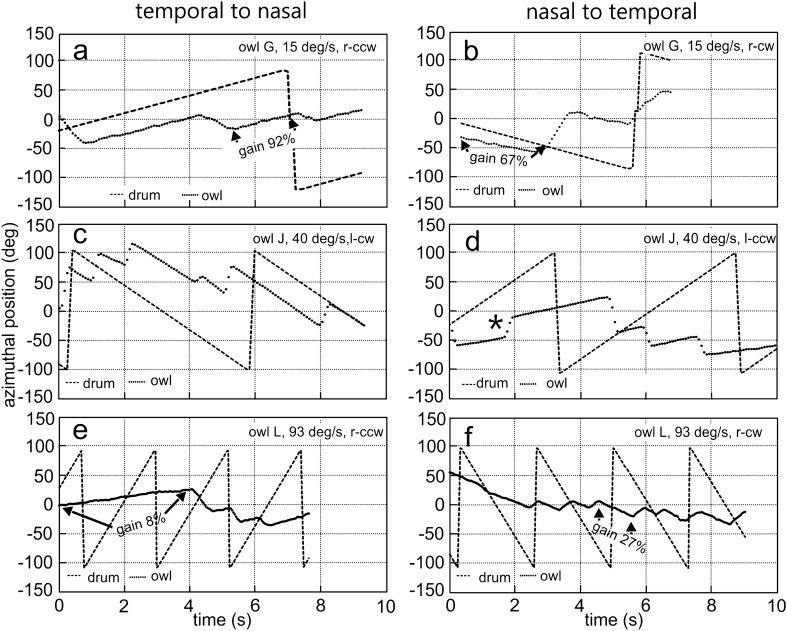
Examples of monocular OCRs of adults. For three velocities: **a**, **b** 15 deg/s, **c**, **d** 40 deg/s, **e**, **f** 93 deg/s the reactions to stimulation in T–N (**a**, **c**, **e**) and N–T directions (**b**, **d**, **f**) are shown. Note that the examples in **a**, **b** and **e**, **f** are from owls G and L, respectively, for which no data were shown in Fig. 3. The stimulation conditions are shown in the inset as well. Gain values are plotted for selected slow-phase segments in **a**, **b**, **e**, and **f**. Other specifications as in the legend to Fig. 3. Note the differences in the gains for stimulation in the T–N and N–T directions

**Fig. 7:**
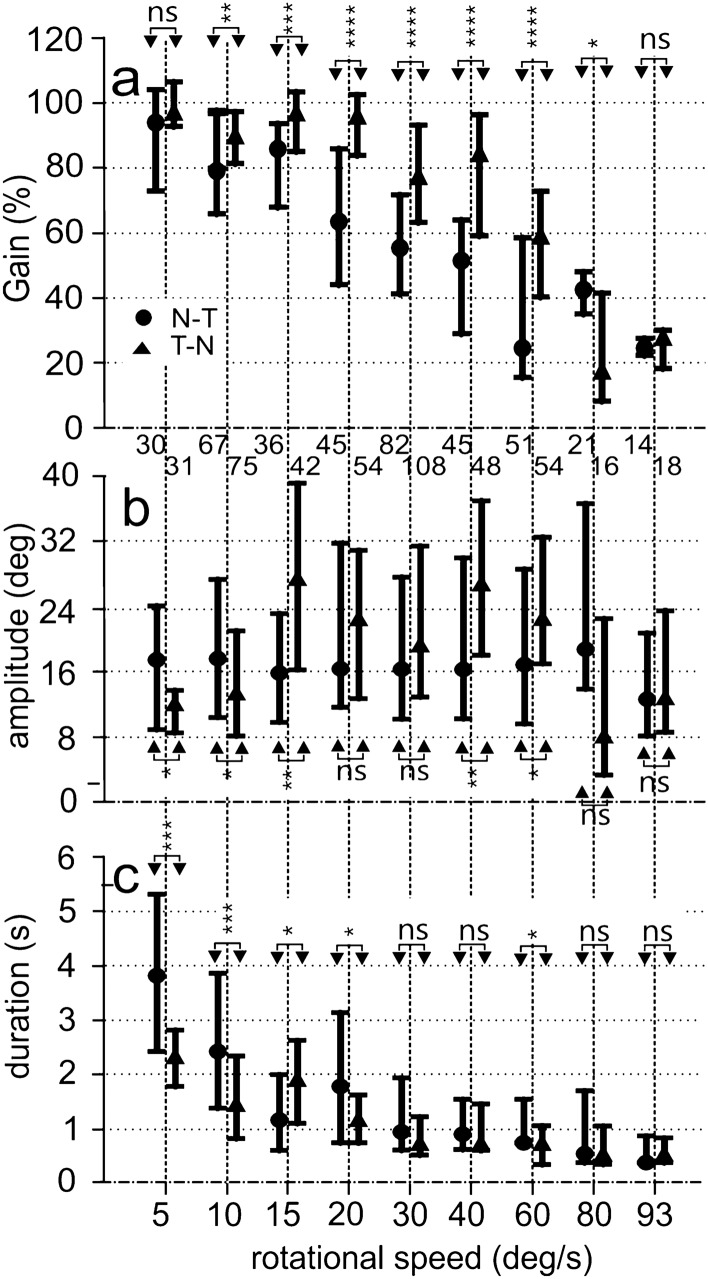
Specific characteristics of monocular OCRs. **a** Gains. **b** Turning amplitude. **c** Duration. N–T: circles, T–N: triangles. Shown are the median values and the first and third quartiles. Results of statistical analysis (*p*-values) are indicated: *ns* not significant, *p < 0.05, **p < 0.01; ***p < 0.001; ****p < 0.00001. Gains are higher for T–N than for N–T stimulation in the middle range of velocities. Amplitudes vary a lot, duration decreases with increasing velocity

In the following, we first briefly describe the typical behavior of the owls during the recording sessions as observed by watching the birds (see video in supplements), then present data from binocular stimulation (Figs. 3, 4, 5) that serves as reference for the subsequent monocular data (Figs. 4, 6, 7), and finally compare both data sets (Tables 1 and 2).

**Table 1:** Comparison of binocular and T–N data

vel^a^	#bino^b^	#T–N^b^	Gain			Amplitude			Duration		
			*U*	*z*-score^c^	*p*	*U*	*z*-score^c^	*p*	*U*	*z*-score^c^	*p*
5	52	31	539	− 2.5088	0.01208	649	− 1.47327	0.14156	734	− 0.67309	0.50286
10	66	75	1784,5	2.85097	0.00438	1790	2.822	0.00466	1929.5	2.25185	0.02444
20	61	54	1558.5	− 0.49315	0.62414	1580.5	0.36987	0.71128	1602	− 0.24938	0.80258
30	73	108	3062	2.54342	0.01108	3415.5	1.52113	0.12852	2596	− 0.12457	0.90448
40	63	48	1357	0.9164	0.35758	1364.5	0.875	0.37886	1478.5	− 0.19643	0.84148
60	63	54	1153	2.99341	0.00278	1284.5	2.27445	0.02232	1685.5	0.08201	0.93624

Mann–Whitney *U* tests

^a^vel = velocity in deg/s

^b^#x: number of cases

^c^A positive *z*-score indicates a higher value for binocular stimulation

**Table 2:** Comparison of binocular and N–T data

vel^a^	#bino^b^	#N–T^b^	Gain			Amplitude			Duration		
			*U*	*z*-score^c^	*p*	*U*	*z*-score^c^	*p*	*U*	*z*-score^c^	*p*
5	52	30	742	− 0.36101	0.71884	470.5	− 2.97473	0.00298	455	− 3.12395	0.0018
10	66	67	1138	− 4.82643	0.00001	2053.5	0.70653	0.4777	1918	− 1.3163	0.18684
20	61	45	485,5	5.66639	0.00001	1211	1.02909	0.30302	1078	− 1.87921	0.0601
30	73	82	1023.5	− 7.05839	0.00001	2305	2.46452	0.0139	2591.5	− 1.43749	0.14986
40	63	45	405.5	6.30328	0.00001	817.5	3.73586	0.00018	1237	− 1.12169	0.26272
60	63	51	492.5	6.34566	0.00001	859	4.25703	0.00001	1116.5	− 2.78958	0.00528

Mann–Whitney *U* tests

^a^vel = velocity in deg/s

^b^#x: number of cases

^c^A positive *z*-score indicates a higher value for binocular stimulation

### Observation of owls during recording

During recording, owls were sitting on a perch and could move their head and body freely. They did so frequently. There were periods during which the owls followed the stimulus, interrupted by periods during which the owls re-oriented their vision (see video in supplements). Often the owls looked downwards or upwards. During these periods, the owls partly followed the stimulus, but these sequences could not be analyzed, because often none or only one of the position markers were visible. If both of the markers were visible when the owls looked up- or downwards, the distance between the markers was short, which might have caused large reconstruction errors. Moreover, it was not clear to where the owl directed its vision and attention. Therefore, we only analyzed those sequences during which the head was held approximately horizontal, in other words, head pitch as judged from the videos was within approximately ± 30° and did not change much during a sequence.

### Binocular optocollic responses

Stimulation with a wide-field pattern very reliably elicited the binocular OCR in adult barn owls. The birds showed persisting reactions for all stimulus velocities tested. During the slow-phase segments, the owls consistently rotated the head in the direction of pattern-rotation. In the following, we first present six typical examples (Fig. 3), before we turn to a quantitative analysis (Figs. 4, 5).

The examples shown in Fig. 3 demonstrate that the owl followed the stimulus. If the stimulus turned in the ccw direction, the head also rotated ccw (Fig. 3a–c), and vice versa (Fig. 3d–f). The angular velocity of head rotation during a slow-phase segment was close to constant as is indicated by the almost linear change of head azimuth with time (Fig. 3). A saccadic turn in the opposite direction terminated a slow-phase segment. Very seldom a brief turn in the direction of movement was observed (see * in Figs. 3e, 6d). Taken over the whole duration of a recording sequence, the return saccades had lower amplitudes than the slow-phase segments so that the orientation of the owl’s head at the end of a sequence that typically contained more than one slow-phase segment was, in most cases, more in the direction of stimulus movement than the starting position. Note that in the example shown in Fig. 3c the owl ceased to follow the pattern before the sequence ended as is indicated by the non-linear trace of the head at the end. Head rotation had a velocity that was close to stimulus velocity during the slow-phase segments, especially for low stimulus velocities. This resulted in gains close to 100% (Fig. 3a–d). Gains tended to be lower at higher stimulus velocities (Fig. 3e, f). Typically, the slow-phase segments were longer for low stimulus velocities than for high stimulus velocities (compare Fig. 3a, b with Fig. 3e, f).

Binocular data was mainly obtained with the square wave pattern (376 slow-phase movements), the remaining (11) with the squares. Since a Mann–Whitney *U* test did not show a difference in the gains measured with the two patterns (*U* = 1606; *z* score = 1.262; *p* = 0.208), the data was lumped, and all further analyses are based on all 387 slow-phase movements. For the monocular data, data sets obtained with the two stimulus patterns were compared when responses were based at respective velocities from at least three owls. Since three of four such data sets did not show a significant difference either, also for monocular stimulation the 847 data obtained with the two different patterns were lumped.

The eyes are symmetrically arranged with respect to the midsagittal plane. Therefore, the binocular OCR with ccw or cw rotation should differ only in the direction of the animal's response velocity but not in the value of the gain. Indeed, a difference in gain for stimulation in the ccw or the cw directions could not be detected, if the data obtained with all stimulus velocities were taken into account (Mann–Whitney *U* test, number of cases ccw = 195, number of cases cw = 192; *U* = 17,051, *z* score = 1.516, *p* = 0.129). This held also, if the data of the individual velocities were considered (Fig. 5a).

Before analyzing the data for the individual velocities quantitatively, we checked the distributions of the gains (Fig. 4). Both, the binocular as well as the monocular gains exhibited a skewed distribution. The monocular gains exhibited a higher tail towards 0 gain than the binocular gains. This bore out in 46% of the monocular gain being below 70%, while only 22% of the binocular gains were below this value. Gains > 100% were observed for most stimulus velocities and were especially not restricted to low velocities. The highest gain we measured was 126%. The maximum number of cases was slightly below 100% gain in both distributions. Both distributions had a long tail towards lower gains and a short tail towards higher gains. Since the distributions were skewed, we decided to present medians and quartiles and analyze the data by nonparametric statistics.

For stimulus velocities up to 30 deg/s, the median gains were about 90%, while for 30 and 40 deg/s a small drop was observed (Fig. 5a). For 60 deg/s, the median gain dropped to 70%. The statistical analysis revealed that the gain at 60 deg/s was smaller than the gains at the other velocities (*p* < 0.0029 for 40 deg/s and lower p-values for the other stimulus velocities, right most column in Table S1). The cross-comparisons for the other velocity pairs suggested that, for example, the gain was higher for a stimulus velocity of 10 deg/s than for stimulus velocities of 5, 30, and 40 deg/s, but not for 20 deg/s (Table S1). Moreover, the gain for a stimulus velocity of 20 deg/s was higher than the gain for stimulus velocities of 30 and 40 deg/s (Table S1). Finally, we like to mention that the highest velocity we measured during a slow-phase segments was 70 deg/s.

The response amplitude tended to increase from low to high stimulus velocities (Fig. 5b). Seventy-eight percent of the amplitudes of the slow-phase segments were lower than 40°. The highest amplitude measured was 134 deg. Median amplitude was lowest for a stimulus velocity of 5 deg/s (Fig. 5b). Table S2 in the supplements documents the comparisons for all velocities (i.e. turning amplitudes at a stimulus velocity of 5 deg/s vs turning amplitudes at a stimulus velocity of 10 deg/s, etc.). Note, for example, that the p-value of each test for 5 deg/s with one of the other velocities is below < 0.00001 (upper row in Table S2). As is already implicated in the presentation of the median data in Fig. 5b and indirectly also in Table S2, turning amplitude was positively correlated with stimulus velocity, if all 387 data pairs (turning amplitude, stimulus velocity) were subjected to a correlation analysis (correlation coefficient: 0.2707, *p* < 0.01; linear equation: turning amplitude (deg) = 19.07 + 0.298*stimulus velocity).

The duration of a slow-phase segment also depended on stimulus velocity, with lower velocities eliciting longer durations (Fig. 5c). The median duration dropped from about 2–0.8 s for velocities from 5 to 30 deg/s. For 40 deg/s and 60 deg/s, the median duration stayed at about 0.8 s. The longest slow-phase segment lasted 17.84 s. Table S3 in the supplements documents the comparisons for all stimulus velocities (for a detailed explanation on how Table S3 has to be read, see above). For example, duration for 5 and 10 deg/s was longer that for the other stimulus velocities (top two rows in Table S3). Correlation analysis including all 387 data pairs demonstrated a highly negative correlation between duration and stimulus velocity (correlation coefficient: − 0.4177, *p* < 0.01; linear equation: duration (s) = 3.51–0.048*stimulus velocity).

In summary, binocular stimulation revealed similar to equal high gains for counterclockwise und clockwise stimulation, increase in amplitudes and decrease in durations of the slow-phase segments with stimulus velocity.

### Monocular optocollic responses

Monocular OCRs were in many respects similar to binocular OCRs (compare Fig. 6 with Fig. 3 and Fig. 7 with Fig. 5). This held specifically for the monocular OCR induced by motion of the stimulus in the T–N direction (see section “Comparison of binocular and monocular data” below). For example, the OCR shown in Fig. 6a in reaction to T–N stimulation with 15 deg/s exhibited a similarly high monocular gain as the OCR plotted in Fig. 3a that was recorded under binocular stimulation. By contrast, the monocular gain measured with stimulation in the opposite, N–T, direction at the same velocity was lower (Fig. 6b) (for a quantitative analysis, see below). Differences between the gains measured with T–N and N–T stimulations were higher for a velocity of 40 deg/s (Fig. 6c, d). For a velocity of 93 deg/s, monocular gains were low for both stimulus directions (Fig. 6e, f).

The monocular gains were generally high, reaching medians slightly below 100% for velocities up to 30 deg/s (Fig. 7a). This held specifically for the gains recorded with T–N stimulation. For higher velocities, the gains were lower, and the medians were only about 20% at the highest velocity tested, 93 deg/s (Fig. 7a).

The monocular gains upon stimulation in the T–N direction were larger than those in the N–T direction for stimulus velocities ranging from 10 to 80 deg/s (Fig. 7a, Table S4). By contrast, the high gains measured for a stimulus velocity of 5 deg/s for T–N and N–T stimulation were not statistically different (Fig. 7a). Likewise, at the highest stimulus velocity tested (93 deg/s) the low gains of N–T and T–N responses were not statistically different (Fig. 7a).

**Fig. 8:**
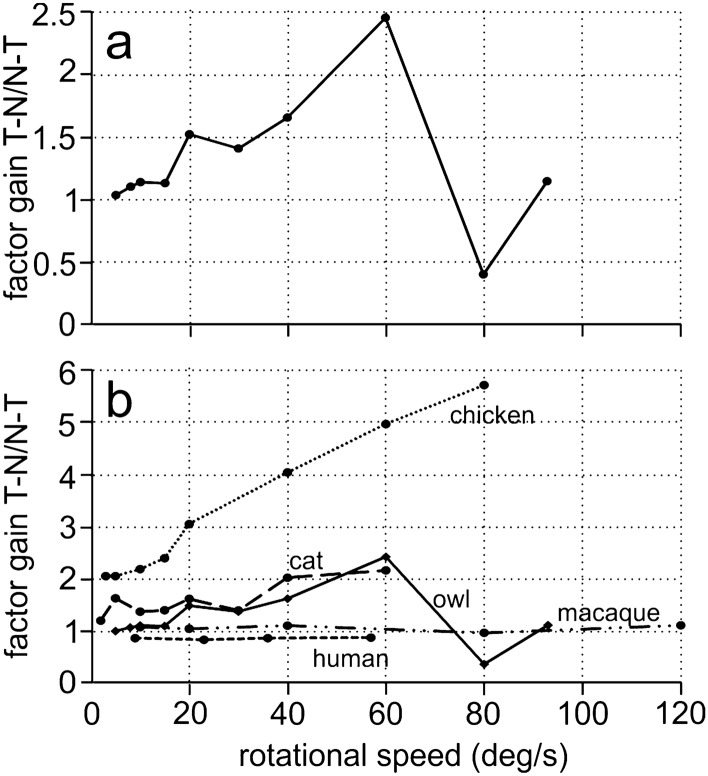
Comparison of monocular OKN in different species. **a** The factor T–N gain/N–T gain is plotted for the barn owl data from this study. **b** The data from the owl are compared with those from the cat (Schweigart and Hoffman 1988), the chicken (Wallman and Velez 1985), the macaque (Distler et al. 1999), and man (van den Berg and Collewjin 1988). Note that the OCR in barn owls is less symmetrical than that of humans and macaques, but more symmetrical than that in the chicken and for most velocities also in the cat

The differences may be quantified by computing the factor gain T–N/gain N–T for each velocity separately (Fig. 8a). This calculation shows that the factors are close to one for low velocities (5, 8, 10, 15 deg/s), but also at the highest velocity tested (93 deg/s). In the medium range (20, 30, and 40 deg/s) of the tested stimulus velocities, the factor amounts to around 1.5. The maximum was 2.45 for 60 deg/s. The data point at 80 deg/s, with a factor of 0.41, is based on few data only (see Table S4). As implicated by the differences in the gains, the factors are statistically different for stimulus velocities from 10 to 80 deg/s, but not for 5 deg/s and 93 deg/s (Table S4).

Turning amplitude was slightly different between N–T and T–N stimulation. Amplitude tended to be larger for T–N responses than for N–T responses in the medium velocity range and lower for the low velocities. However, overall, the variability was high as demonstrated by the large differences between the values at the third and first quartiles of the distributions (Fig. 7b). Correlation analysis demonstrated a weak, but significant positive relation for both T–N and N–T stimulation (N–T: 395 data points, correlation coefficient: 0.01, *p* < 0.01; linear equation: turning amplitude (deg) = 21.33 + 0.034*stimulus velocity; T–N: 452 data points, correlation coefficient: 0.05, *p* < 0.01; linear equation: turning amplitude (deg) = 21.57 + 0.005*stimulus velocity).

The durations of the slow-phase segments dropped from longer values at low stimulus velocities to shorter values at high stimulus velocities (Fig. 7c). The durations of the N–T responses were significantly longer than the durations of the T–N responses at low stimulus velocities (5, 10 and 20 deg/s), with a reverse effect for 15 deg/s (Fig. 7c). A significant difference could not be detected for higher stimulus velocities (30, 40, 80 and 93 deg/s), with an exception of 60 deg/s for which the N–T responses were longer than the T- N responses (Fig. 7c). Correlation analysis demonstrated a significant negative relation for both T–N and N–T stimulation (N–T: 395 data points, correlation coefficient: -0.39, p < 0.01; linear equation: duration (s) = 2.65–0.027*stimulus velocity; T–N: 452 data points, correlation coefficient: − 0.41, p < 0.01; linear equation: duration (s) = 1.89–0.019*stimulus velocity).

Overall, monocular OCRs to T–N stimulation showed higher gains than OCRs to N–T stimulations. By contrast, there were only minor differences in turning amplitude and slow-phase segment duration.

### Comparison of binocular and monocular data

Response characteristics of binocular and monocular OCRs were similar. The gains were higher for binocular stimulation than for T–N stimulation at three velocities (10, 30, 60 deg/s) (Table 1). For 5 deg/s, the reverse was true, with no significant differences for 20 and 40 deg/s (Table 1, Figs. 5, 7). The comparison of turning amplitudes yielded significantly higher amplitudes for binocular stimulation for 10 and 60 deg/s, with no difference for the other velocities (Table 1). Finally, the comparison of the slow-phase durations only yielded a difference at 10 deg/s, where duration was longer for binocular stimulation than for T–N stimulation (Table 1).

Gains for all stimulus velocities, apart from 5 deg/s, were higher for binocular stimulation than for N–T stimulation (Table 2, Figs. 5, 7). Turning amplitudes for binocular stimulation were higher than for N–T stimulation for 30, 40, and 60 deg/s, lower for 5 deg/s and not statistically different for 10 and 20 deg/s (Table 2, Figs. 5, 7). The duration of the slow-phase movements was longer for N–T stimulation than for binocular stimulation for 5 and 60 deg/s, but not statistically different for the other velocities tested (10, 20, 30, 40 deg/s) (Table 2, Figs. 5, 7).

Overall, conspicuous differences between reactions to binocular and to monocular stimulation occurred only for the gains. Specifically, gains for N–T stimulation were clearly lower than for binocular stimulation while T–N gains were close to the binocular values.

## Discussion

We shall discuss our data in the following with respect to the methods used by us and by others, with respect to optocollic, optomotor and optokinetic responses of other animals, including man, and end with an outlook.

### Methodological considerations

Owls compensated for wide-field stimuli with head rotations just as mammals compensate with eye movements. The experiments revealed a high gain of both the binocular and monocular OCRs, especially for low velocities. Pigeons react in a similar way as owls do, although they have larger eye-movement capabilities than owls (Gioanni et al. 1981; Gioanni 1988; Türke et al. 1996). However, pigeons do not make major use of their eye-movement capability, if they can freely move their head (Haque and Dickman 2004).

As mentioned above, the untrained owls moved their head and body a lot while standing on the perch. Periods of fixation were sometimes short, sometimes longer. Thus, the stimulus situation was less standardized than in OKR studies where the head of the animal is fixed or restricted to rotation around a central axis only. The possibility to move improves gaze stabilization for stimulus velocities higher than 20 deg/s (Maurice et al. 2006), and increases overall performance of the optokinetic nystagmus (Wallman 1993). Consequently, median gain in barn owls was high, close to 100%, at low stimulus velocities (Fig. 5a), even when one eye was occluded (Fig. 7a). Values were comparable to the “standing condition” in pigeons (Maurice et al. 2006). Gain in barn owls was larger than in actively standing pigeons in the same set-up (Türke et al. 1996) which suggests high OCR-reactivity in barn owls. In particular, vestibular self-stimulation during head rotation did not seem to interfere with OCR up to 30 deg/s, but may have contributed to a drop in gain towards high velocity stimuli so that the range of the effective velocities was narrower in the OCR of owls than in the OKR of macaques (Distler et al. 1999). We know of no behavioral study on the vestibular-collic reflex in barn owls.

Closed-loop gain as defined in Eq. (1) showed a wide distribution with some gains being higher than 100% (Fig. 4). Closed-loop gains larger than 100% are not expected in a simple feedback system, because they suggest a reversal of the sign of the retinal slip speed. They, thus, deserve a discussion. Gains > 100% were also reported in other studies (e.g. Wallman and Velesz 1985; Gioanni and Vidal 2012). One factor that has to be taken into account when interpreting this seemingly over-compensation of the wide-field visual stimulus is the arbitrary definition of gain. In Eq. (1), we compared head and drum velocity; however, the owl's optokinetic system may be driven by a correlation-mechanism to extract pattern motion, similar to pigeons (Türke et al. 1996). Since this mechanism does not extract exact drum velocity, but a signal that depends on spatial structure and contrast of the pattern, in particular higher harmonics may change the perceived velocity and may result in faster head rotation. Another aspect is the frequent eccentric head position of the owls in our setup. Since the frontal orientation of the eyes restricts the field of view in this species to about 190° (Knudsen 1982), an eccentric head position will lead to distortions of the perceived stimulus depending on the distance to the frontal wall of the drum and orientation of the owl’s head. A further aspect is that the closed-loop gain as defined in Eq. (1) does not reflect the internal processing: an "internal signal" that adds to the reflexive head movement (like a command variable in control theory) could alter the closed-loop gain of the system, and, thus, also result in gains > 100%.

Further, the responses of the owl itself bear features that interfere with the definition in equation one. Independently of the owl’s head position at the onset of a slow-phase segment, a mandatory additional eccentricity occurs during the rotation in the slow-phase segment, because of anatomical reasons the head of the barn owl always translates while it rotates (Ohayon et al. 2006; Krings et al. 2017). Furthermore, eye movements [maximum 3° in horizontal direction (Du Lac and Knudsen 1990)] may change gains for slow-phase amplitudes. However, if the owls behaved like pigeons, eye movements would not be expected to contribute much to the gains (Gianni 1988; Hague and Dickman 2004). Finally, part of the gains > 100% may also be due to noise, both in the owls’ behavior and in the reconstruction. In summary, many factors that we did not control might influence the perceived stimulus velocity and lead to gains > 100%. However, gains larger than 100% need not signify a retinal-slip speed in the opposite direction.

### Optocollic, optokinetic, and optomotor responses in other animals

Wide-field movement is a very strong stimulus that elicits compensatory eye or head rotations in practically all animals that possess an elaborated visual sense. While most animals show a response to a moving visual wide-field stimulus, there are major variations between species (for reviews see Huang and Neuhauss 2008; Masseck and Hoffmann 2009). With binocular stimulation, gains typically approach 100%, at least at moderate velocities. The responses elicited by monocular stimulation, however, vary considerably. For reasons of simplicity, we sort the monocular responses into three categories: (1) In many vertebrates the optomotor response to stimulation in the N–T direction is practically absent or of very low gain (for reviews see Huang and Neuhauss 2008; Masseck and Hoffmann 2009). (2) A reaction to stimulation in N–T direction is observed, but it is much weaker than that occurring to stimulation in the T–N direction (factor T–N/N–T > 1.2, e.g. rabbit: Collewijn1969; pigeon: Gioanni 1988; chicken: Wallman and Velez 1985, Fig. 8b; cat: Schweigart and Hoffmann 1988, Fig. 8b; mice: Kretschmer et al. 2017). 3) The reactions in both stimulation directions are equivalent like in humans (Fig. 8b; van den Berg and Collewijn 1988) and macaques (Fig. 8b; Distler et al. 1999). The data on the barn owl presented here puts this species between the second and third category, as a symmetric OCR was observed for low velocities, while weak asymmetry occurred for middle-range velocities (Fig. 8).

The reason for the differences between species has been a matter of much debate (e.g. Huang and Neuhauss 2008; Masseck and Hoffmann 2009). Masseck and Hoffmann (2009) considered several hypotheses like frontal orientation of the eyes, decussation pattern of retinal fibers, foveation, eye position and resulting binocular overlap, lifestyle and degree of independence of eye movement. The conclusion of their analysis was that “no universally valid theory can be suggested for all vertebrate classes to explain symmetry versus asymmetry of monocular OKR”. Even if there is no unifying theory, arguments for one or the other hypothesis may be advanced. Our results add some pieces of information to the data. Barn owls have frontally oriented eyes and a large binocular overlap (Willigen et al. 1998; Nieder and Wagner 2001) but no fovea (Oehme 1961), an almost total decussation of retinal fibers at the midbrain level, but a fusion of the information from the two eyes through the supraoptic chiasm in the forebrain (Karten et al. 1973). Moreover, barn owls are predators with a specialization for sound localization, but use visual information whenever possible (Harmening and Wagner 2011; Wagner et al. 2013), and they possess a coupled accommodation but an independent pupillary reflex (Schaeffel and Wagner 1992). Thus, the data from barn owls presented here seem to rather complicate than solve the implications of the data available from other species. Nevertheless, the fact that owls have binocular vision and a symmetrical horizontal OCR for at least low velocities supports, in our view, the argument that a symmetrical rotational OCR is a feature of animals with frontally placed eyes. However, as pointed out above, this is not an argument that can be used in a causal sense for every case of symmetric responses, because also some lateral-eyed animals show a more or less symmetric response (Masseck and Hoffmann 2009). It would also be interesting to study the vertical OCR in owls and find out whether it is asymmetric as in many frontal-eyed animals, including humans (van den Berg and Collewijn 1988). If we restrict our consideration to birds, most lateral-eyed species exhibit an asymmetric response. An exception may be hummingbirds (Goller and Altshuler 2014; Gaede et al. 2016; Goller et al. 2019). Hummingbirds use optic flow to control their delicate motion when feeding. There is a uniform distribution of direction sensitive cells in the nucleus lentiformes (Gaede et al. 2016), suggesting that the OCR may be symmetric. However, to our best knowledge, this has not been measured.

### Outlook

We present here basic data on the OCR of adult barn owls and show that the OCR of owls is phenomenologically much closer to the OKR of primates than to the OCR of its closer relatives, birds (Fig. 8). Many more data are necessary to substantiate this claim. For example, we have not trained the owls, and, thus, head and body movements affected the responses. Due to the frequent movements, we could not discriminate between early and late OCRs components. It might also be interesting to study whether a "dynamic fixation" or "look"-OCR can be elicited and under which conditions this might be evoked. In mammals, the optokinetic response is driven by a subcortical network that is influenced by inputs from the visual cortex (Grasse et al. 1984; Wallman 1993; Distler et al. 2002). The neuronal circuit underlying the OCR in owls is not well known. We have some preliminary data demonstrating a bilateral projection from the visual Wulst to several midbrain and diencephalic nuclei (Wirth and Wagner 2019), but more data are necessary to unravel the neuronal circuit or to show whether response properties of optomotor neurons in barn owls are similar to those in frontal-eyed mammals, similar to what Wylie et al. (1994) demonstrated for saw-whet owls. Moreover, in primates, the symmetry is not present in very young babies, but develops with age (Distler et al. 1999). We shall present data on the development of OCR in baby barn owls separately (Wagner et al., in preparation).

## Supplementary Information

Below is the link to the electronic supplementary material.

Supplementary file1 (DOCX 13 kb)

Supplementary file2 (DOCX 12 kb)

Supplementary file3 (DOCX 12 kb)

Supplementary file4 (DOCX 12 kb)

Supplementary file5 (AVI 8802 kb)

## Abbreviations

ccw: Counterclockwise
cw: Clockwise
deg: Degrees
N–T: Nasal to temporal
OCR: Optocollic response
OKR: Optokinetic response
OMR: Optomotor response
T–N: Temporal to nasal
