# Supplementary material for: Optocollic responses in adult barn owls (Tyto furcata)
Source: J Comp Physiol A Neuroethol Sens Neural Behav Physiol. 2021 Nov 23;208(2):239–51. doi: 10.1007/s00359-021-01524-z (PMC8934767; doi:10.1007/s00359-021-01524-z)
Supplement: Supplementary file 1 — Supplementary file1 (DOCX 13 kb) [file 359_2021_1524_MOESM1_ESM.docx]

Table S1 Mann-Whitney U-Test binocular all: gain across stimulus velocities

| V | 10* | 20* | 30* | 40* | 60* |
| --- | --- | --- | --- | --- | --- |
| 5 | 2.056; -333.5; 0.038 | 0.786; -1449; 0.430 | 1.162; 1665.5; 0.246 | 1.705; 1334; 0.087 | 4.557; 826.5; <0.00001 |
| 10 |  | 1.329; 1737; 0.184 | 3.393; 1604; 0.0007 | 3.812; 1269.5; 0.0001 | 6.835; 729; <0.00001 |
| 20 |  |  | 2.098; 1756.5; 0.036 | 2.452; 1430.5; 0.0143 | 6.358; 849; <0.00001 |
| 30 |  |  |  | 0.687; 2141.5; 0.490 | 3.904; 1404.5; 0.0001 |
| 40 |  |  |  |  | 2.976; 1374; 0.0029 |

* Shown are the z-score, U and p, for number of cases see Tables 1 and 2, positive z-score indicates higher value for velocity noted in left column
