# Supplementary material for: Optocollic responses in adult barn owls (Tyto furcata)
Source: J Comp Physiol A Neuroethol Sens Neural Behav Physiol. 2021 Nov 23;208(2):239–51. doi: 10.1007/s00359-021-01524-z (PMC8934767; doi:10.1007/s00359-021-01524-z)
Supplement: Supplementary file 2 — Supplementary file2 (DOCX 12 kb) [file 359_2021_1524_MOESM2_ESM.docx]

Table S2 Mann-Whitney U Test binocular all: amplitude across stimulus velocities

| V | 10* | 20* | 30* | 40* | 60* |
| --- | --- | --- | --- | --- | --- |
| 5 | 4.345; 914;<0.00001 | 4.853; 743;<0.00001 | 5.738; 753; <0.00001 | 6.437; 492;<0.00001 | 6.797; 428; <0.00001 |
| 10 |  | 1.235; 1756.5;0.215 | 1.820; 1977; 0.069 | 3.475;1341;0.0005 | 3.798;1272.5;0.0001 |
| 20 |  |  | 0.447; 2126; 0.6527 | 1.904; 1540; 0.057 | 2.267; 1467.5; 0.023 |
| 30 |  |  |  | 1.730; 1920.5; 0.084 | 2.237;1786.5; 0.025 |
| 40 |  |  |  |  | 0.278; -1927; 0.779 |

* Shown are the z-score, U and p, for number of cases see Tables 1 and 2, positive z-score indicates higher value for velocity noted in top row
