# Supplementary material for: Optocollic responses in adult barn owls (Tyto furcata)
Source: J Comp Physiol A Neuroethol Sens Neural Behav Physiol. 2021 Nov 23;208(2):239–51. doi: 10.1007/s00359-021-01524-z (PMC8934767; doi:10.1007/s00359-021-01524-z)
Supplement: Supplementary file 3 — Supplementary file3 (DOCX 12 kb) [file 359_2021_1524_MOESM3_ESM.docx]

Table S3 Mann-Whitney U Test binocular all: duration across stimulus velocities

| V | 10* | 20* | 30* | 40* | 60* |
| --- | --- | --- | --- | --- | --- |
| 5 | 0.312; 1656.5, 0.749 | 3.269; 1018; 0.001 | 5.412; 816; <0.00001 | 5.440; 669.5; <0.00001 | 6.572; 468; <0.00001 |
| 10 |  | 3.359; 1316.5; 0.001 | 6,040; 976.5; <0.00001 | 6.097; 784.5, <0.00001 | 7.268; 536; <0.00001 |
| 20 |  |  | 2.033; 1771; 0.0424 | 2.097; 1501.5; 0.036 | 3.706; 1179.5, 0.0002 |
| 30 |  |  |  | 0.159; 2262.5; 0.873 | 2.350; 1760.5, 0.0188 |
| 40 |  |  |  |  | 2.215; 1530; 0.026 |

* Shown are the z-score, U and p, for number of cases see Tables 1 and 2, positive z-score indicates higher value for velocity noted in left column
