# Supplementary material for: Optocollic responses in adult barn owls (Tyto furcata)
Source: J Comp Physiol A Neuroethol Sens Neural Behav Physiol. 2021 Nov 23;208(2):239–51. doi: 10.1007/s00359-021-01524-z (PMC8934767; doi:10.1007/s00359-021-01524-z)
Supplement: Supplementary file 4 — Supplementary file4 (DOCX 12 kb) [file 359_2021_1524_MOESM4_ESM.docx]

Table S4 Mann-Whitney U-Test: T-N vs N-T monocular gains

| V | #T-N^1^ | #N-T^1^ | U | z-score | p |
| --- | --- | --- | --- | --- | --- |
| 5 | 30 | 31 | 351.5 | 1.63 | 0.1031 |
| 10 | 67 | 75 | 1837 | 2.76 | 0.00578 |
| 15 | 36 | 42 | 427,5 | 3,288 | 0.001 |
| 20 | 45 | 54 | 427,5 | 5,53 | 0.00001 |
| 30 | 82 | 108 | 2297 | 5.675 | 0.00001 |
| 40 | 45 | 48 | 464 | 4.732 | 0.00001 |
| 60 | 51 | 54 | 751.5 | 4.007 | 0.00001 |
| 80 | 21 | 16 | 95.5 | 2.282 | 0.0271 |
| 93 | 14 | 18 | 101.5 | 0.912 | 0.36282 |

1: number of cases
